# Supplementary material for: Appendiceal microbiome in uncomplicated and complicated acute appendicitis: A prospective cohort study
Source: PLoS One. 2022 Oct 14;17(10):e0276007. doi: 10.1371/journal.pone.0276007 (PMC9565418; doi:10.1371/journal.pone.0276007)
Supplement: S1 Fig — (PDF) [file pone.0276007.s001.pdf]

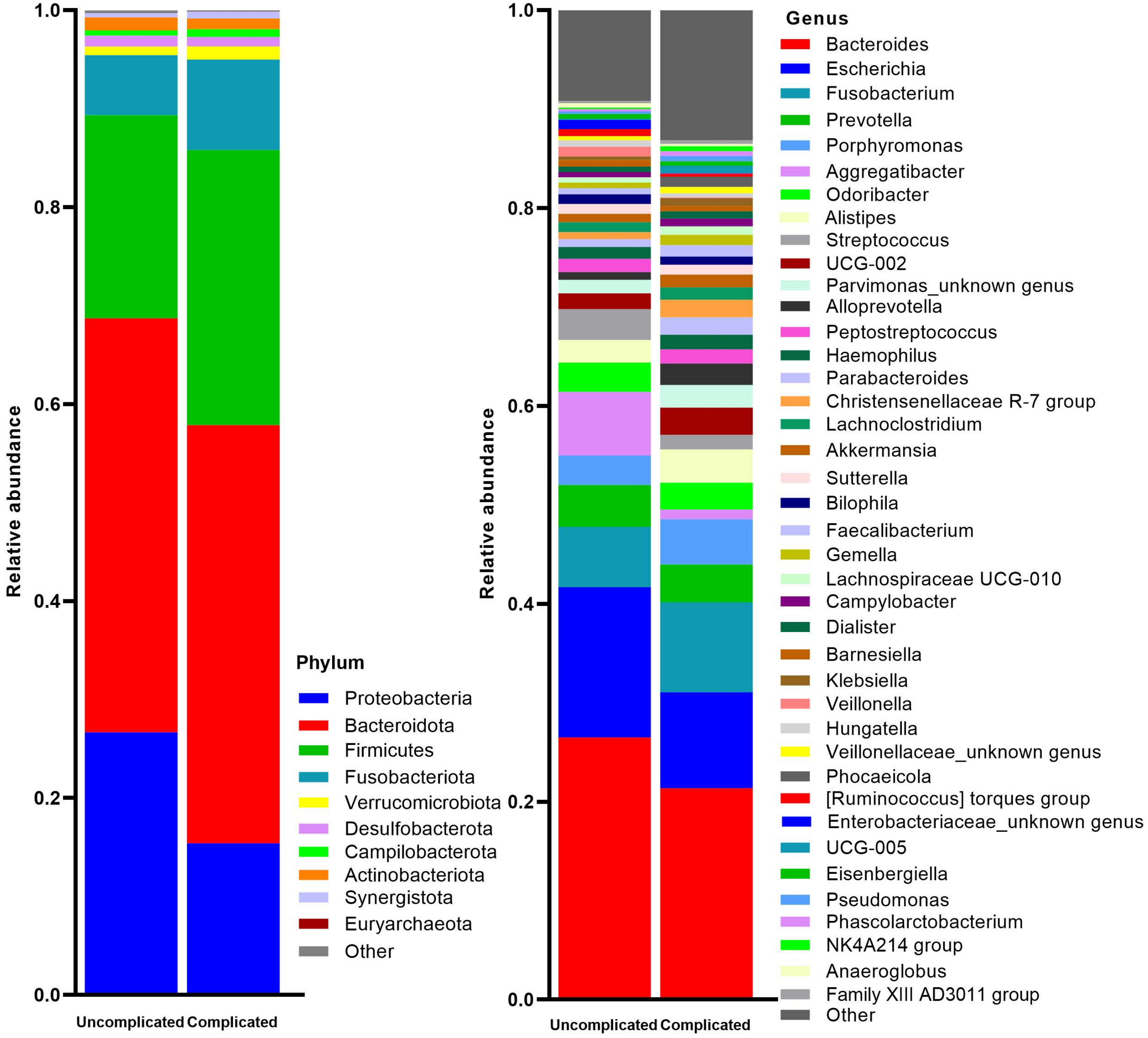

S3 Fig. Appendiceal microbiome in phylum and genus level in uncomplicated and complicated acute appendicitis. Stacked barplots indicate the mean relative abundance of phyla and genera grouped by appendicitis severity.
