# Supplementary material for: Appendiceal microbiome in uncomplicated and complicated acute appendicitis: A prospective cohort study
Source: PLoS One. 2022 Oct 14;17(10):e0276007. doi: 10.1371/journal.pone.0276007 (PMC9565418; doi:10.1371/journal.pone.0276007)
Supplement: S1 Table — (PDF) [file pone.0276007.s002.pdf]

S1Table. Differentially abundant bacterial species and genera between uncomplicated and complicated appendicitis.

Species	BaseMean	Log2fold change	p-value	p-value adjusted
Veillonella parvula	31,4	6,4	6,5E-13	5,6E-11
Haemophilus unknown species	34,6	5,7	4,9E-08	3,2E-06
Veillonella unknown species	12,3	5,3	4,3E-05	1,4E-03
Campylobacter concisus	3,6	5,3	8,7E-04	9,2E-03
Aggregatibacter aphrophilus	406,4	4,6	3,7E-04	5,0E-03
family Enterobacteriaceae unknown species	44,5	4,6	3,0E-04	4,8E-03
Streptococcus unknown species	272,7	2,7	1,2E-04	2,8E-03
Bacteroides fragilis	8268,5	2,4	3,9E-04	5,0E-03
UCG-005 unknown species	202,2	-2,4	3,6E-04	5,0E-03
Family XIII UCG-001 unknown species	4,8	-2,4	6,1E-05	1,8E-03
order Clostridia UCG-014 unknown species	43,4	-2,5	8,8E-04	9,2E-03
Roseburia unknown species	8,1	-2,7	5,1E-04	6,0E-03
Paludicola unknown species	3,2	-2,7	3,7E-04	5,0E-03
Alistipes obesi	5,5	-2,7	8,9E-04	9,2E-03
Subdoligranulum unknown species	26,9	-2,8	1,4E-05	5,0E-04
Defluviitaleaceae UCG-011 unknown species	1,5	-2,8	1,4E-04	2,8E-03
Faecalibacterium unknown species	21,9	-3,1	7,7E-05	2,0E-03
[Eubacterium] siraeum group unknown species	4,4	-3,3	2,8E-04	4,8E-03
family Christensenellaceae unknown species	30,3	-3,6	6,1E-07	2,6E-05
Dialister unknown species	38,9	-4,0	4,2E-04	5,1E-03
Porphyromonas endodontalis	146,5	-4,8	2,6E-04	4,8E-03
Bacteroides faecis	681,8	-5,4	7,7E-08	4,0E-06
Phocaeicola abscessus	22,9	-7,1	1,4E-04	2,8E-03
Phascolarctobacterium unknown species	26,9	-10,5	6,7E-14	8,7E-12
**Genus**	**BaseMean**	**Log2fold change**	**p-value**	**p-value adjusted**
Aggregatibacter	2849,8	5,3	1,4E-06	1,6E-04
Veillonella	35,0	5,1	2,0E-12	4,5E-10
Unknown genus from enterobacteriaceae	56,8	4,9	1,1E-04	4,0E-03
Oscillibacter	60,3	-2,2	4,7E-05	2,1E-03
Family XIII UCG-001	4,4	-2,2	1,5E-04	4,0E-03
Subdoligranulum	25,1	-2,6	3,3E-05	1,8E-03
Paludicola	3,0	-2,6	3,7E-04	8,2E-03
Defluviitaleaceae UCG-011	1,4	-2,7	2,4E-04	6,0E-03
Unknown genus from Christensenellaceae	22,3	-3,2	4,0E-06	3,0E-04
Phocaeicola	22,7	-7,0	1,4E-04	4,0E-03

Positive values in Log2fold change indicate higher abundance in uncomplicated appendicitis compared to complicated appendicitis
