# Supplementary material for: Appendiceal microbiome in uncomplicated and complicated acute appendicitis: A prospective cohort study
Source: PLoS One. 2022 Oct 14;17(10):e0276007. doi: 10.1371/journal.pone.0276007 (PMC9565418; doi:10.1371/journal.pone.0276007)
Supplement: S2 Table — (PDF) [file pone.0276007.s003.pdf]

S2 Table. Relative abundance of species in uncomplicated and complicated appendicitis.

Species	Mean relative abundance uncomplicated %	Mean relative abundance complicated %
Escherichia unknown species	15,19	9,69
Bacteroides fragilis	12,28	7,42
Bacteroides dorei	1,91	2,88
family parvinomas unknown species	1,36	2,30
Porphyromonas endodontalis	1,11	1,41
Fusobacterium unknown species	4,88	6,93
Bacteroides faecis	0,92	2,15
Bacteroides unknown species	6,90	5,34
Alistipes onderdonkii	1,30	1,56
Odoribacter splanchnicus	1,62	1,24
Peptostreptococcus stomatis	1,35	1,43
Haemophilus parainfluenzae	0,76	1,49
Bacteroides thetaiotaomicron	1,70	1,19
Odoribacter unknown species	1,35	1,41
Akkermansia muciniphila	0,53	1,04
Phocaeicola abscessus	0,00	1,04
Aggregatibacter aphrophilus	3,80	0,18
Fusobacterium nucleatum	0,95	1,18
UCG-002 unknown species	1,60	2,73
Aggregatibacter segnis	2,04	0,63
Pseudomonas unknown species	0,28	0,55
Bilophila unknown species	1,00	0,83
Streptococcus unknown species	2,39	0,72
Prevotella nigrescens	0,67	1,66
Gemella unknown species	0,36	0,79
Prevotella intermedia	0,36	0,85
Porphyromonas gingivalis	0,52	0,63
Dialister invisus	0,52	0,42
Lachnospiraceae UCG-010 unknown species	0,52	0,85
Porphyromonas unknown species	1,36	2,56
Alistipes putredinis	0,28	0,54
Faecalibacterium prausnitzii	0,59	1,04
family Veillonellaceae unknown species	0,42	0,64
Bacteroides vulgatus	0,62	0,65
Christensenellaceae R-7 group unknown species	0,72	1,79
Streptococcus anginosus	0,71	0,71
Fusobacterium necrophorum	0,00	0,66
Sutterella wadsworthensis	0,73	0,74
Parabacteroides merdae	0,18	0,37
Bacteroides xylanisolvens	0,80	0,21
Alistipes finegoldii	0,46	0,48
Lachnoclostridium unknown species	0,95	1,20
Bacteroides uniformis	0,45	0,37
Phascolarctobacterium faecium	0,20	0,33
Eisenbergiella tayi	0,36	0,27
Bacteroides ovatus	0,50	0,30
Alloprevotella unknown species	0,62	1,96

Akkermansia unknown species	0,32	0,27
Prevotella unknown species	2,15	1,10
NK4A214 group unknown species	0,15	0,50
family Christensenellaceae unknown species	0,09	0,37
Solobacterium unknown species	0,25	0,31
Hungatella hathewayi	0,59	0,37
Tannerella forsythia	0,17	0,29
Campylobacter unknown species	0,38	0,52
Aggregatibacter unknown species	0,58	0,16
[Ruminococcus] torques group Unknown	0,71	0,30
UCG-005 unknown species	0,15	0,76
Alloprevotella tannerae	0,16	0,20
Veillonella parvula	0,52	0,03
Lawsonella clevelandensis	0,08	0,24
family Enterobacteriaceae unknown species	0,92	0,05
Veillonella unknown species	0,48	0,03
Fusobacterium varium	0,00	0,21
Parabacteroides distasonis	0,32	0,49
Actinomyces unknown species	0,38	0,10
Parabacteroides unknown species	0,26	0,87
Alistipes unknown species	0,18	0,60
Klebsiella unknown species	0,34	0,72
Eikenella corrodens	0,30	0,07
S5-A14a unknown species	0,25	0,32
Prevotella micans	0,30	0,00
Anaeroglobus unknown species	0,42	0,23
Gemella morbillorum	0,21	0,24
Prevotella oris	0,42	0,07
Anaerotruncus unknown species	0,05	0,13
Incertae Sedis unknown species	0,11	0,21
family Lachnospiraceae unknown species	0,25	0,35
Fretibacterium fastidiosum	0,12	0,11
Barnesiella unknown species	0,67	0,52
UBA1819 unknown species	0,10	0,11
Bacteroides cellulosilyticus	0,08	0,12
Comamonas kerstersii	0,18	0,00
Eggerthella lenta	0,14	0,09
Flavonifractor plautii	0,10	0,12
family Tannerellaceae unknown species	0,03	0,12
Bacteroides kribbi	0,05	0,12
Bacteroides caccae	0,08	0,12
[Eubacterium] coprostanoligenes group unknown species	0,11	0,23
Intestinimonas butyriciproducens	0,05	0,12
Tuzzerella unknown species	0,09	0,14
Family XIII AD3011 group unknown species	0,24	0,38
Lawsonella unknown species	0,02	0,13
Firmicutes order DTU014 unknown species	0,03	0,17
Fusobacterium periodonticum	0,21	0,00
Roseburia inulinivorans	0,11	0,12
Acidaminococcus intestini	0,00	0,14

Campylobacter curvus	0,02	0,22
Dialister unknown species	0,03	0,29
Subdoligranulum unknown species	0,01	0,15
Filifactor alocis	0,03	0,15
Oscillibacter unknown species	0,08	0,11
Sutterella unknown species	0,25	0,24
Coprococcus unknown species	0,02	0,15
Eisenbergiella unknown species	0,10	0,17
Coprobacter unknown species	0,12	0,08
family Oscillospiraceae uknown species	0,09	0,15
UCG-003 unknown species	0,07	0,14
family Ruminococcaceae unknown species	0,12	0,23
Prevotella baroniae	0,20	0,00
Atopobium unknown species	0,30	0,07
Cloacibacillus evryensis	0,22	0,05
Agathobacter unknown species	0,07	0,16
Bacteroides pyogenes	0,00	0,22
[Eubacterium] eligens group unknown species	0,07	0,13
order Clostridia UCG-014 unknown species	0,03	0,21
Flavonifractor unknown species	0,10	0,03
Intestinimonas unknown species	0,10	0,18
Selenomonas unknown species	0,10	0,25
Cloacibacillus unknown species	0,05	0,12
Lachnospiraceae NK4A136 group unknown species	0,08	0,21
[Eubacterium] nodatum group unknown species	0,05	0,13
Blautia unknown species	0,06	0,14
family Peptoniphilus unknown species	0,11	0,10
Clostridium sensu stricto 1 unknown species	0,42	0,02
Sanguibacteroides unknown species	0,13	0,01
Haemophilus unknown species	0,45	0,01
Clostridia vadinBB60 group unknown species	0,03	0,11
Eggerthella unknown species	0,12	0,04
Serratia unknown species	0,13	0,11
Eikenella unknown species	0,17	0,07
Fretibacterium unknown species	0,05	0,17
Prevotellaceae UCG-001 unknown species	0,17	0,02
Raoultella planticola	0,10	0,00
Tannerella unknown species	0,00	0,10
Lachnospiraceae UCG-004 Unknown	0,05	0,18
Catonella unknown species	0,11	0,04
Butyricimonas unknown species	0,22	0,20
Phascolarctobacterium unknown species	0,02	0,12
Faecalibacterium unknown species	0,03	0,12
Campylobacter concisus	0,10	0,01

Species composing more than 0.1% in each group are in the table
