# Supplementary material for: Appendiceal microbiome in uncomplicated and complicated acute appendicitis: A prospective cohort study
Source: PLoS One. 2022 Oct 14;17(10):e0276007. doi: 10.1371/journal.pone.0276007 (PMC9565418; doi:10.1371/journal.pone.0276007)
Supplement: Attachment — Submitted filename: renamed_b9133.docx [file pone.0276007.s004.docx]

**COMMENTS – Manuscript PONE-D-22-06832**

Title: “Appendiceal microbiome in uncomplicated and complicated acute appendicitis: a prospective cohort study (MAPPAC)”

**Important note: This review pertains only to ‘statistical aspects’ of the study and so** **‘clinical aspects’ [like medical importance,** **relevance of the study,** **‘clinical significance and implication(s)’ of the whole study, etc.] are to be evaluated [should be assessed] separately/independently. Further please note that any ‘statistical review’ is generally done under the assumption that (such) study specific methodological [as well as execution] issues are perfectly taken care of by the investigator(s). This review is not an exception to that and so does not cover clinical aspects {however, seldom comments are made only if those issues are intimately / scientifically related & intermingle with ‘statistical aspects’ of the study}. Agreed that ‘statistical methods’ are used as just tools here, however, they are vital part of methodology [and so should be given due importance].**

**COMMENTS:** In my opinion, since this study was a single-center arm of a multicenter MAPPAC (Microbiology Appendicitis Acuta) trial, the study should be treated as having different ‘Study design and methodology’. Therefore, quoting previously published protocol describing these [Study design and key methods have been reported previously (30)] is not correct. This quoted ‘study protocol is for the ‘MAPPAC trial’ and is definitely excellent, however, since this publication is not ‘one of the series’, giving sufficient details regarding ‘Study design and methodology’ are expected. That protocol is for two arms two clinical trials but the present one is single arm study. How can the ‘Study design and methodology’ be same? Therefore, is that really relevant quoting previously published protocol? In ‘Results’ section saying that “Altogether 308 patients were enrolled in the MAPPAC study between April 11, 2017, and March 29, 2019; Figure 1 shows the study flow” do you think has any meaning/relevance?

May I request you to make a small change in title of study [“Appendiceal microbiome in uncomplicated and complicated acute appendicitis: a prospective cohort study (MAPPAC)”] because ‘MAPPAC’ is another trial. This is only a suggestion (as inclusion of the term ‘MAPPAC’) may cause confusion and average reader like me, will take the present study as MAPPAC. You may not follow this as this one is my subjective opinion [please free to take appropriate decision].

Since this study is based on ‘single-arm design’, may I further request to read the following [which is pasted from one standard textbook on ‘Research Methodology’]:

For a pilot study it is alright to have ‘single-arm design’, or it is alright when that is the only possibility’, however, It is very essential to keep the limitations in mind while interpreting results. Further, note that a classical/ideal clinical trial/study needs/requires a concurrently {but similarly} handled/treated appropriately selected/chosen control/comparison parallel group/arm.

Note further that “Inferential statistics (i.e., hypothesis testing + estimation of CI) is built on the population model [which means the underlying assumption is that there is/are population(s) and we are dealing with random sample(s) drawn from that/those population(s)]. Although in clinical trial (involving at least two groups) we do not really deal with random samples (generally a non-probabilistic convenience sampling), ‘allocation’ to treatment groups is ‘randomly’ done which enable us to evoke the population model and we can use inferential statistics safely. But when there is only one group (so that there is no question of random allocation), with ‘non-random’ selection, it may be questionable to use inferential statistics even if you have two measurement sets as ‘pre-post’ or use ‘internal grouping for comparison” [like here uncomplicated acute appendicitis compared to complicated appendicitis but this is an ‘internal grouping’].

I am sure that these learned authors already know these things, however, it is very essential to keep the limitations in mind while interpreting results {note that I am not asking you to change the study design}.

What exactly do you mean by ‘excluded due to failed library preparation (n=26)’? Please note that any ‘regression models / regression techniques’ are not originally developed for head-to-head between group comparison(s), [Alpha diversity analyses were performed using standard linear regression models with alpha diversity measure (Chao1, Shannon entropy or number of observed species) as the response variable]. Nevertheless, limitations of the study highlighted on page 14 are highly appreciated. However, as pointed out in ‘important note’ above “This review pertains only to ‘statistical aspects’ of the study and so ‘clinical aspects’ should be assessed separately/independently [one should carefully consider/look at the clinical implications of the study].

One last minor doubt, refer to page 15 where you said “To our knowledge, this is the first prospective study with a large patient cohort and standardized clinical definitions of appendicitis severity comparing the microbial composition of the appendix (may be true) in adult patients between uncomplicated and complicated acute appendicitis” and quoted reference number 14 for this. My doubt is ‘What evidence this reference provided?’ to say you so or ‘what in this reference is indicative of this fact?’.

Except these minor points, in my opinion, this article/manuscript is good and needs a small amount of re-vision (which is quite possible). After minor revision, this manuscript may be accepted [provided clinical implications of these findings/results are valuable].
